# Plantar pressure distribution during treadmill walking in comfort shoes with PLA(Poly Lactic Acid) resins

**DOI:** 10.1186/1757-1146-7-S1-A123

**Published:** 2014-04-08

**Authors:** Seung-Bum Park, Kyung-Deuk Lee, Dae-Woong Kim, Jung-Hyeon Yoo, Kyung-Hun Kim

**Affiliations:** 1 Footwear Biomechanics Team, Footwear Industrial Promotion Center, Busan, Korea

## 

In the framework of environmentally friendly processes and products, poly lactic acid(PLA) represents the best polymeric substitutes for various petropolymers because of its renewability, biodegradability, biocompatibility and good thermomechanical properties [1]. The purpose of this study was to analyze foot pressure distribution of PLA materials in functional shoes. Comfort is an important aspect in footwear. Footwear comfort has an influence on injury [2,3]. The development of new materials is considered as the important point for manufacturing functional shoes [4].

Ten healthy female(mean height: 159.8 cm, mean body mass: 54.8 kg, mean age 20.8 yrs.) participated in this study. All subjects were free of lower extremity pain, history of serious injuries or operative treatment, or subjective symptoms interfering with walking.

**Figure 1:**
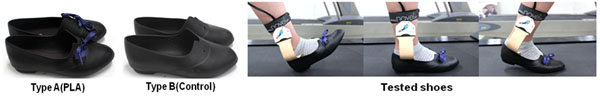
The two different shoes conditions: Type A(PLA), Type B(Control), Size 245mm

The subjects were required to walking(3.2km/h) for treadmill. Each subject wore two different shoes, type A(PLA) and Type B(control)(figure 1) during walking. The PEDAR^®^-X insole system(Novel GmbH, Germany) was used to measure the foot pressure and force. Pressure distribution data(contact areas, maximum force, peak pressure, maximum mean pressure) was collected with pressure device at a sampling rate of 100Hz. The feet were divided into four regions: foot(Total), forefoot, midfoot, rearfoot.

**Figure 2:**
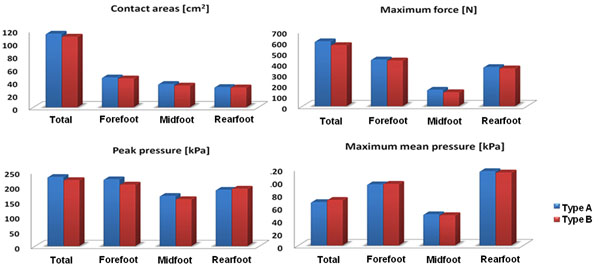
Comparison of foot pressure.

Results of foot pressure distribution date show that(figure 2) contact area increased by 4% in the type A compared to type B, Also, maximum mean pressure decreased by 5%. However, peak force increased by 6%, and peak pressure increased by 5% as well. AS a result PLA resins may be helpful in decreasing overall pressure in foot therefore provide better comfort in foot.
